# Multiscale Coronary Arterial Network Generation and Hemodynamics Using Patient-Specific Fractional Myocardial Blood Volume

**DOI:** 10.3390/bioengineering12111274

**Published:** 2025-11-20

**Authors:** Mostafa Mahmoudi, Arutyun Pogosyan, Amirhossein Arzani, Kim-Lien Nguyen

**Affiliations:** 1 Division of Cardiology, David Geffen School of Medicine at UCLA and VA Greater Los Angeles Healthcare System, Los Angeles, CA 90095, USA; mmahmoudi@mednet.ucla.edu (M.M.); arutyunpogosyan@mednet.ucla.edu (A.P.); 2 Department of Bioengineering, University of California, Los Angeles, CA 90095, USA; 3 Department of Mechanical Engineering, University of Utah, Salt Lake City, UT 84112, USA; amir.arzani@sci.utah.edu; 4 Scientific Computing and Imaging Institute, University of Utah, Salt Lake City, UT 84112, USA; 5 Department of Radiological Sciences, David Geffen School of Medicine at UCLA, Los Angeles, CA 90095, USA

**Keywords:** ischemic heart disease, microvascular disease, arterial network generation, adaptive constrained constructive optimization, myocardial perfusion, fractional myocardial blood volume, magnetic resonance imaging, digital heart

## Abstract

Ischemic heart disease (IHD) is the leading cause of death worldwide. Although 90% of the intramyocardial blood volume resides in the microvasculature, clinical imaging methods cannot visualize the microvascular coronary network in vivo, and non-invasive hemodynamic estimates overlook patient-specific microcirculatory contributions. Herein, we present a multiscale framework to extend the epicardial coronary tree and generate 1D microvascular networks in the myocardium based on ferumoxytol-enhanced magnetic resonance coronary imaging and fractional myocardial blood volume (fMBV) maps. Synthetic arterial networks were constructed from MRI data belonging to three swine, four healthy volunteers, and one IHD patient using a modified multistage, adaptive constrained constructive optimization approach. Hemodynamic simulations were performed in synthetic arterial networks. Morphological parameters were compared with empirical models. In 126 arterial networks (*n* = 6000 terminal segments per subject per seed; six seeds per coronary vessel), the morphometry was strongly correlated with empirical data (*r* > 0.87), with low variability (*CoV* < 0.01) across multiple rounds of network simulations. Mixed-effects models and a Dynamic Time Warping analysis confirmed robustness and repeatability. In the IHD patient, simulated arterial networks (*n* = 15) reproduced tissue-dependent morphological and functional signatures consistent with coronary autoregulation in scar and hypoperfused tissues. The findings establish an early potential for patient-specific microvascular network synthesis and hemodynamic simulations from MRI data.

## 1. Introduction

Diagnosis, monitoring, and therapeutic decisions in ischemic heart disease (IHD) rely on the depiction of the coronary anatomy and function. Although the vast majority of the intramyocardial blood volume resides in the microcirculation, high-resolution clinical imaging systems cannot depict the microvascular network due to spatiotemporal limitations. To overcome the limited resolution in clinical imaging systems and provide a more comprehensive depiction, patient-specific myocardial perfusion data can be integrated with computational fluid dynamic analysis to enable automated myocardial arterial network generation and simulations of hemodynamic behavior.

Non-invasive cardiac perfusion stress testing methods, including nuclear or first-pass gadolinium perfusion magnetic resonance imaging (MRI), adopt a framework that is designed for the detection of epicardial coronary stenosis [1] rather than microvascular disease. Although dynamic stress CT myocardial perfusion imaging (CT-MPI) and coronary CT angiography (CTA) provide whole-heart myocardial perfusion along with anatomic coronary images, CT-MPI is timed to capture the transit of iodinated contrasts during the systolic cardiac phase, whereas coronary CTA is timed to capture anatomic coronary images during mid-diastole [1]. This discordance in the cardiac phase alignment during the image acquisition makes it difficult to spatially and physiologically correlate anatomic coronary narrowing with myocardial perfusion. Moreover, both iodinated and gadolinium-based contrast agents extravasate almost immediately after injection, requiring a correction for leakage in quantitative perfusion models.

Recently, researchers have proposed the use of ferumoxytol-enhanced (FE) MRI in combination with a two-compartment water exchange model to map the fractional myocardial blood volume (fMBV) as a marker for hypoperfusion [2,3,4]. Ferumoxytol (Feraheme, Azurity Pharmaceuticals, Woburn, MA, USA) is an iron supplement that can be used as a pure intravascular MRI contrast agent to sensitize the intravascular space to changes in blood volume, enabling the accurate estimation of the fMBV. With increased oxygen demand, the body’s autoregulatory system will increase capillary recruitment, leading to increased fMBV, and in areas of microvascular dysfunction, there is decreased fMBV thought to be related to decreased capillary density or dysfunctional microvessels [5]. Unlike CT-MPI and coronary CTAs, both FE-MRI fMBV maps and whole-heart coronary MRAs are acquired during the steady-state distribution of ferumoxytol and during mid-diastole.

In this study, we propose to develop a synthetic patient-specific framework for the automatic generation of a one-dimensional arterial network in the myocardium based on perfusion data derived from FE-MRI fMBV maps and FE coronary MRA. Consistent with principles of hemodynamic efficiency, whereby the blood volume distribution throughout a solid organ is determined by the local metabolic demand, we hypothesize that FE-MRI fMBV maps, reflecting the blood volume distribution, along with FE coronary MRA will enable the accurate and reliable automatic generation of the myocardial arterial network based on a modified adaptive constrained constructive optimization (CCO) approach. Existing methods for automatic myocardial arterial network generation include fractal models [6], space-filling methods that encompass optimization algorithms [7], and continuous models with porous media flow simulations [8]. Of the space-filling methods, the adaptive CCO approach can be tailored to reflect the physiological and anatomical variations in the myocardial arterial network [9]. Although existing adaptive CCO approaches excel at capturing intricate network details and accommodating patient-specific physiological variations, they have not been tailored for the coronary circulation. Despite decades of progress in hemodynamic modeling techniques, the generation of a large, physiologically sound, and patient-specific vascular network that accurately supplies blood to the ventricular myocardial segments remains challenging. Our contributions are three-fold: (1) the introduction of a modified framework that constructs adaptable, multiscale, and physiological 1D arterial networks in subject-specific myocardium; (2) the incorporation of FE-MRI fMBV maps to inherently account for the microcirculation’s blood flow autoregulation mechanism; and (3) the computation of reproducible hemodynamic indices at any location across the synthetic arterial network, which is crucial for studying the full spectrum of ischemic heart disease that requires the consideration of the microcirculation.

## 2. Materials and Methods

This article is a revised and expanded version of a paper entitled “Synthesis of Arterial Networks from Myocardial Blood Volume Maps”, which was presented at the 2024 ASME SB3C Summer Bioengineering Conference in Lake Geneva, WI, USA, on 11–14 June 2024. All animals were treated in accordance with the Guidelines for the Care and Use of Laboratory Animals, the Animal Welfare Act, and the National Institutes of Health. The UCLA Animal Research Committee (ARC-2017-015) approved the animal studies. The human subject research was conducted in accordance with the Declaration of Helsinki. All patients provided written or electronic informed consent prior to participation. The Institutional Review Board at UCLA (IRB-19-2097) and the Veterans Health Administration (VA0004; IRBNet# 1615882) approved the human research studies.

### 2.1. Study Design

This study was designed to develop and validate a patient-specific and non-invasive framework to synthetically construct coronary arterial networks from MRI. The approach integrates FE MRI to obtain both coronary MRA and fMBV maps, which serve as inputs to a modified adaptive constrained constructive optimization algorithm for generating 1D vascular trees. Validation was performed in swine (*n* = 3) and healthy volunteers (*n* = 4), where 126 arterial networks (6000 terminal segments each) were generated using six random seedings per coronary tree and evaluated against empirical morphometric data. Computational simulations of pulsatile blood flow were performed for all generated microvascular networks, and reproducibility across seedings was assessed using mixed-effects models and waveform similarity metrics. Finally, in a patient with IHD, we demonstrate the framework’s ability to recapitulate tissue-dependent changes in morphological and functional parameters of microvasculature and its consistency with coronary autoregulation in areas of ischemia, scar, and remote myocardium.

### 2.2. Subject Characteristics

Three healthy Yorkshire swine (female, 20–33 kg), four healthy female human volunteers (age 35 ± 10.2 years), and one IHD patient with myocardial ischemia and scar were included. For the swine subjects, intramuscular ketamine (10 mg/kg), midazolam (1 mg/kg), and inhaled 1–2% isoflurane vaporized with 100% oxygen were used to achieve sedation and anesthesia. Intravenous rocuronium (2.5 mg/kg/h) was administered to immobilize the diaphragm during anesthesia and MRI. Hemodynamic and electrocardiographic vital signs were monitored throughout all procedures. This HIPAA-compliant study recruited both healthy volunteers and patients with IHD age ≥ 18 years to undergo an FE-MRI.

### 2.3. Magnetic Resonance Image Acquisition and Processing

We performed multi-dose FE-MRI T1 mapping on healthy swine and human subjects using the Modified Look-Locker Inversion (MOLLI) recovery sequence on a 3.0 T (Prisma and Skyra, Siemens, Erlangen, Germany) clinical magnet as previously described [4]. Using the 5(3)3(3)3 MOLLI recovery sequence, we acquired 7–10 left ventricular (LV) short axis T1 maps from base to apex. A balanced steady-state free precession (SSFP) readout with the following parameters was utilized: FOV = 300 × 240 mm, matrix size = 192 × 156, TR = 338 ms, TE = 1.01 ms, slice thickness = 8 mm, pixel bandwidth = 1085 Hz, flip angle = 35°, minimum T1 = 100 ms, and TI increment 80 ms. Diluted ferumoxytol (Feraheme, Azurity Pharmaceutical, Woburn, MA, USA) was infused for a cumulative dose of 4.0 mg/kg (ferumoxytol increment range 0.0–4.0 mg/kg). An in-house two-compartment water exchange model with three parameter fitting was used to generate pixel-wise fMBV maps [2,3] from multi-slice, 2D short axis T1 maps covering the entire ventricular myocardium from base to apex. Using 3D Slicer v5.2.1 [10], we segmented each pixel-wise fMBV map based on the AHA 16-segment model [11].

The 4D MUSIC pulse sequence [12] was used to image the coronaries of the swine subjects, whereas the whole-heart coronary 3D MRA (WIP 1111, Siemens Healthcare, Erlangen, Germany) was used for human subjects. Both were acquired at steady-state ferumoxytol enhancement (4 mg/kg). Representative technical parameters for 4D MUSIC were as follows: TR/TE of 3.0/1.1 ms, flip angle of 25°, pixel bandwidth of 800 Hz/pixel, and isotropic spatial resolution of 1.1 mm^3^. The whole-heart coronary MRA is a free-breathing, gradient echo sequence with Dixon imaging for fat suppression and inversion recovery preparation pulses. Representative parameters for the whole-heart coronary MRA sequence were as follows: TR = 985 ms, TE = 1.39 ms, flip angle = 15–18°, and isotropic spatial resolution of 1.1 mm^3^.

### 2.4. Vascular Network Generation

To realistically represent perfusing epicardial coronary arteries and microvasculature, we designed an adaptive, multistage CCO algorithm capable of generating an arterial network initialized from the epicardial coronary arteries and then extending into the myocardium without going beyond the endocardial surface. Inputs into the algorithm included the initial 1D epicardial coronary tree, myocardial perfusion territories represented as spatial myocardial point clouds, corresponding fMBV values, and weight functions. Utilizing the input data, the framework dynamically adjusts bifurcation placements and vessel orientations to reflect the branching gradients observed in physiological conditions. This adaptation allows the synthetic network to maintain a balance between minimizing the vascular volume and supplying blood to various myocardial segments while adhering to the structure of the myocardial geometry.

Arterial segments in our algorithm were modeled as cylindrical tubes, with Poiseuille’s law governing flow as incompressible Newtonian fluid under laminar, steady-state flow conditions. Murray’s law with a power coefficient of 3 determined the relationship between parent and daughter segment radii. fMBV-derived perfusion data were used to ensure that the terminal flow rates accurately reflected the myocardial blood supply, and the density of microvascular network follows the autoregulation mechanism captured by the fMBV maps.

Laplace equation-derived weights were used in the objective function to compute the network’s total weighted volume. The benefit of having a weight function in the computational domain is two-fold: (1) to assist the algorithm in creating a gradient in the number of branching segments from epicardium to endocardium, which more closely matches known physical properties of coronary vessels and (2) to ensure that the vascular network conforms to the patient-specific myocardium geometry. These parameters satisfy the underlying biological assumption that vascular tree growth is geometrically dictated by the organ it perfuses.

The staged growth mechanism involved five phases for each major coronary arterial tree (left anterior descending [LAD], left circumflex [LCX], and right coronary artery [RCA]): (1) Epicardial Completion—five terminal segments per myocardial segment were generated to extend the initial tree. (2) Epicardial Growth—ten terminal segments per myocardial segment were added to fill the gaps on the epicardial surface. (3) Endocardial Access—fifty terminal arteries were generated to enable deeper extension into the mid-myocardium and growth towards the endocardial surface. (4) Epicardial Surface Refinement—one hundred terminal segments were added to refine the epicardial network. (5) Terminal Segment Distribution—remaining terminal segments were designed to distribute fully, covering each myocardial perfusion territory based on fMBV maps.

The arterial segment lengths were calculated based on the number of terminal segments in each perfusion territory, myocardial segment volume, and the stage of arterial network generation. Terminal nodes were randomly placed in each myocardial segment and connected to 10 closest arterial segments, with bifurcation locations optimized to minimize the tree volume. The resulting bifurcation with the smallest arterial network volume was then selected. Bifurcations distribute closer to the epicardial surface to mirror the physical properties of coronary vascular perfusion. The optimization function adhered to several constraints: segment lengths were required to have twice the segment diameter and a dynamic maximum length; bifurcation angles were restricted to a pre-defined range; a dynamic bifurcation diameter symmetry coefficient was enforced; and new or modified segments could not intersect existing arterial segments or the endocardial surface. During the first stage of network generation, arterial segments were constrained to avoid overlapping. These constraints ensured physiologically sound, patient-specific network growth within the myocardium. See Supplementary Material S2 for parameter specification.

**Figure 1:**
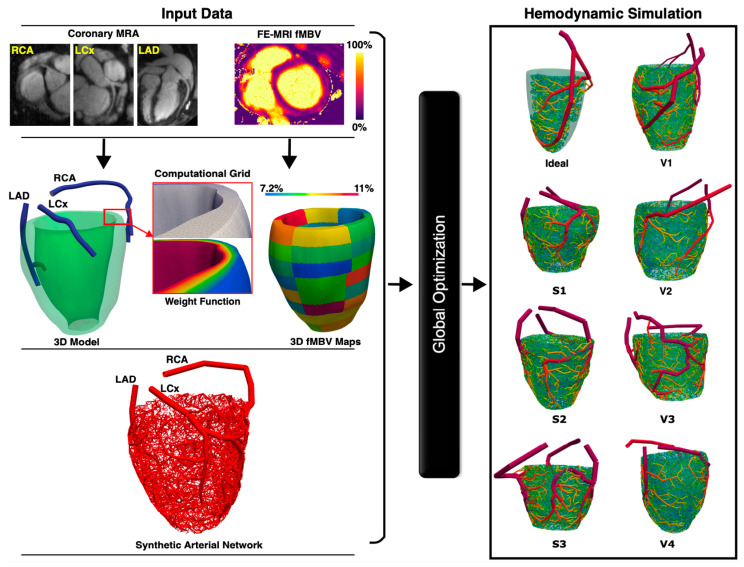
Overview of synthetic arterial network generation and hemodynamic simulations. The framework generates 1D synthetic arterial networks from FE coronary MRA and fMBV maps, which are introduced into a global optimization of arterial diameters (Video S1). The hemodynamic environment is then simulated for ideal myocardium (Ideal), swine (S1–S3), and volunteer (V1–V4) subjects. FE, ferumoxytol-enhanced; fMBV, fractional myocardial blood volume; LAD, left anterior descending; LCx, left circumflex; RCA, right coronary artery; MRA, magnetic resonance angiography; and MRI, magnetic resonance imaging.

A total of 2000 terminal arterial segments were used for each major coronary arterial tree (*n* = 141 synthetic arterial trees from ideal myocardium *n* = 6, swine subjects *n* = 54, healthy volunteers *n* = 72, and IHD patient *n* = 15). The diameter of the most proximal arterial segment (root) of the synthetic arterial network was calculated from the MRA, whereas flow rates and resistances across the network were used to calculate the diameters of each arterial segment in the synthetic network. The FE-MRI fMBV maps were used to define segmental blood flow percentages (SBFPs). SBFP is computed as the average segmental fMBV multiplied by the myocardial volume of each segment, normalized within each epicardial artery. During tree construction, the sum of terminal flows supplying each sub-segment was enforced to equal its allocated share of the artery inflow, based on SBFPs, which served as patient-specific proportional constraints linking myocardial volume and flow distribution. Our automatic arterial network generation was designed to be consistent with principles of hemodynamic efficiency, whereby vascular networks rise to accommodate blood volume distribution governed by local tissue metabolic demands (Figure 1).

Several factors were critical for the automatic arterial network generation based on FE-MRI fMBV maps. First, our modified adaptive CCO approach [9] has a multistage component to ensure that larger arterial segments are on the epicardial surface while the microvascular network perfuses the myocardium. Second, the adaptive component enables simultaneous generation of shorter microvascular segments as the network grows, while updating the geometric and hemodynamic parameters based on the segmental fMBV maps (Video S1).

### 2.5. Hemodynamic Simulation

To evaluate the functionality of the synthetic networks, 1D pulsatile blood flow was simulated for all subjects and seeds. Global optimization was applied prior to each simulation to tune deformable arterial segment diameters, ensuring the mean standard error between synthetic and FE-MRI fMBV-derived flow distributions remained below $10^{-8}$. A general physiological flow rate waveform was prescribed at the root segment of each coronary arterial tree [13,14], and the Olufsen material model was considered for the arterial walls [15]. Distal outlets were prescribed with a fixed perfusion pressure of 80 mmHg, consistent with CCO-based coronary morphometry studies [9]. Intramyocardial pressure (IMP) was not explicitly modeled. At resting end-diastolic cardiac phase, the IMP ranges from approximately 5 mmHg in the epicardium to 20 mmHg in the endocardium [16], which represents a relatively constant offset and does not alter the relative segmental flow distribution. A 20% perturbation in distal pressure was imposed to evaluate the sensitivity of SBFP (see Section 2.10). Hemodynamic simulations used a time step of 0.001 s over 10 cardiac cycles to reach periodic steady state, with a convergence tolerance of 10^−6^ for flow and pressure residuals. See Supplementary Material S2 and S3 for details of numerical solver configuration and pipeline runtime.

### 2.6. Modeling in Idealized Myocardium

For idealized myocardium, we generated six arterial networks using different initial seedings. The LAD artery territory was considered, and a patient-specific 3D model of the LAD was generated from coronary CTA images. The LAD perfusion territory was segmented using the AHA’s 16-myocardial segment model, and the SBFPs were calculated using an empirical model. The morphology of the constructed synthetic trees was compared against Kassab’s empirical model [17], and the geometrical features of the networks were studied. We also solved 1D pulsatile blood flow simulations for each synthetic arterial network. The blood flow rate waveforms and flow resistances at three distal epicardial arterial segments were compared across different seedings. The resulting synthetic SBFPs were compared against those derived from the empirical model.

### 2.7. Subject-Specific Modeling

The 3D models of myocardium were extracted from whole-heart coronary MRA for swine and from multi-slice cine images for human subjects. Segmental fMBV values from MRI were mapped onto the 3D model of the left ventricle, and the volume of blood supplying each left ventricular segment was calculated based on the ratio of average fMBV values in different myocardial segments. Three-dimensional models of the LAD, LCx, and RCA were constructed in Simvascular v2021.06 [18] and then converted to one-dimensional models using the software’s reduced order modeling feature. The morphology of the constructed healthy synthetic trees was compared against Kassab’s empirical model [17], and the geometric features of the networks were studied. One-dimensional pulsatile blood flow simulations were performed for each synthetic arterial network. The blood flow rate waveforms and flow resistances at three distal epicardial arterial segments were compared across different seedings.

### 2.8. Arterial Network Generation and Subject-Specific Modeling in a Patient with Ischemic Heart Disease

To investigate the performance of our proposed framework in several myocardial tissue types, we selected a 74-year-old patient with prior myocardial infarction status post coronary stenting of the LAD vessel with progressive complaints of chest pain on exertion. The patient had undergone stress cardiac positron emission tomography (PET) with Rubidium-82 tracer, late gadolinium enhancement (LGE) MRI, and FE-MRI. Five microvascular networks with 2000 terminal segments using 5 random seedings were constructed for each main epicardial coronary artery based on the patient-specific FE-MRI fMBV maps. The average diameter of the arterial network at Strahler order numbers 1, 2, and 3 as well as the vascular tissue flow delivery (μ= % terminal flow/tissue volume) from the synthetic microvascular networks were extracted and compared between different myocardial tissue types (ischemia, scar, and remote). Ischemic tissue was defined as areas with reversible myocardial perfusion defect on PET. Scar tissue was defined as areas with LGE on gadolinium-based MRI. Remote myocardial tissue was defined as regions with no perfusion defect on PET and without LGE on MRI.

### 2.9. Statistical Analysis

The interquartile range (IQR) for the density of vessels per vessel diameter size was calculated as $Q_{25^{th},\;75^{th}}\pm 4.0\left(IQR\right)$. A wider range of four times the IQR was used to show the consistency of resultant terminal diameter density across various seeds. Coefficient of variation (CoV) was used to assess the relative variability of structural and geometrical parameters in the synthetic arterial networks. This metric was of importance in quantifying the consistency of our synthetic arterial trees across various seeds and subjects. Pearson and Spearman correlation coefficients were used to compare the arterial segment diameters and lengths in our synthetic networks with Kassab’s empirical model [17]. Mixed-effects models were employed to analyze the variability in arterial diameters, flow rates, and flow resistance across various seeds, main coronary arteries, and subjects. These models account for both fixed effects (seeds) and random effects (coronary arteries, myocardial segments, and subjects) and provide an analysis of the factors affecting the observed variability. Dynamic Time Warping (DTW) [19] was employed to measure the dissimilarity between flow rate waveforms from different seeds for each subject and artery. This technique aligns flow rate waveforms to minimize the distance between them, allowing for an accurate comparison of their shapes. Coupled with mixed-effect model, DTW was used to quantify the effect of subject, artery, and initial seeds on the variability in flow rate waveforms. This analysis was important for determining the repeatability of the hemodynamic results of our synthetic framework. See Supplementary Material S5 for complete results of statistical analyses.

### 2.10. Uncertainty Quantification

To test the robustness of the SBFP-based flow allocation across myocardial segments, we performed steady-state 1D simulations in the LAD network of subject V3 (Seed 1) while varying four physiological parameters within ±20% of their baseline values: (i) blood viscosity, (ii) wall stiffness in the Olufsen model, (iii) distal pressure offset, and (iv) inlet flow rate. The resulting segmental SBFP distributions were compared with baseline using the root-mean-square deviation and coefficient of variation. See Supplementary Material S2 for physiological values.

## 3. Results

### 3.1. Testing in Idealized Arterial Network

**Figure 2:**
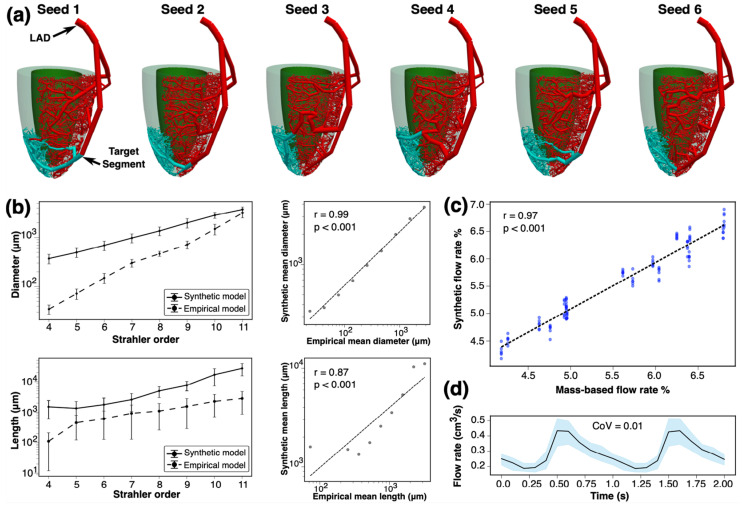
Validation of synthetic arterial network in idealized myocardium. (**a**) Synthetic left anterior descending (LAD) arterial tree from six different initial seeds. Teal-colored vessels represent the sub-network supplying blood to the apical septum. (**b**) Comparison of synthetic arterial diameters and lengths vs. Kassab’s empirical model grouped by Strahler order number. (**c**) Comparison of synthetic and mass-based coronary blood flow percentages. (**d**) Blood flow rate waveforms at the target segment supplying blood to the apical septum. Shaded areas represent the flow rate variation between different seeds.

For an idealized myocardium, six synthetic arterial networks generated from random initial seedings in the LAD territory showed excellent accuracy and reproducibility (Figure 2). Relative to the arterial segment diameter and length in Kassab’s empirical model, our synthetic networks exhibited comparable features based on their Strahler order number [17]. The Strahler order number represents the order of each segment in a bifurcating system [17], whereby terminal segments have a Strahler order number of one. If two daughter arteries with the same order meet, the order of the parent segment increases by one. Otherwise, the maximum order between the daughters is assigned to the parent segment. The Strahler order numbers in the synthetic trees were shifted such that the Strahler order for the root artery, i.e., the most proximal arterial segment, matched Kassab’s Strahler order. The Spearman correlation between synthetic and empirical values of arterial segment diameters (r = 0.99) and lengths (r = 0.87) suggested a high degree of accuracy in replicating the microvascular topology (all *p* < 0.001). There was low variability with a high consistency in the diameters of terminal vessels. The CoV for the diameter of the terminal microvasculature and the total volume of the tree across synthetic arterial trees were 0.008 and 0.009, respectively.

The percentage of the coronary blood flow delivered to myocardial segments showed a strong correlation with the flow rate based on the myocardial mass [20] (Spearman r = 0.97, *p* < 0.001, Figure 2c). Compared to the mass-derived reference segmental blood flow rate, our synthetic network exhibited a similar flow rate distribution, with a higher flow rate in the mid and apical segments. When comparing mass-based vs. synthetically derived segmental flow rate percentages, the CoVs for basal, mid, and apical segments were 0.072, 0.09, and 0.046, respectively. The synthetic network achieved a CoV of 0.01 for the mean flow rate in the parent artery and a CoV of 0.008 for the blood flow resistance at the target arterial segment. These findings support the model’s precision and repeatability for simulating coronary hemodynamics.

### 3.2. Validation of Subject-Specific Arterial Network Topology

**Figure 3:**
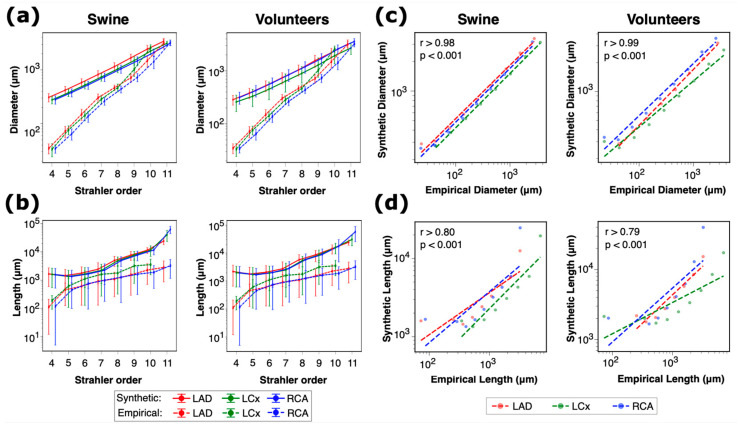
Validation of morphometry from subject-specific synthetic arterial networks. Comparisons for 126 constructed arterial networks are shown (*n* = 6000 terminal segments per subject per seed). Variation in arterial segment diameters (**a**) and lengths (**b**) versus Strahler order for swine and volunteers. Correlation of diameters and lengths between synthetic network and Kassab’s empirical model in swine (**c**) and volunteers (**d**).

We constructed synthetic networks for healthy subjects (*n* = 3 swine, *n* = 4 healthy volunteers) using six different initial seeds. The terminal diameter in each synthetic network reached 200 μm after ~1500 terminal segments in all cases. We compared the morphology of 126 constructed arterial networks to Kassab’s morphometry [17] based on Strahler ordering for the segment diameter and length. The total number of diameter datapoints used for comparison was 291,852 for swine and 387,353 for healthy human volunteers, whereas the total number of segment length datapoints was 212,036 and 283,815, respectively. Differences between the number of diameter and length datapoints were due to our networks being nonbinary; some arterial segments served as extensions of the upstream segment. The variation in segment diameters and lengths for swine and volunteers aligned closely with empirical data (Figure 3a,b). Similar to Kassab’s empirical model [17], we grouped the diameters and lengths of all arterial segments based on the subject type (swine and volunteer) and Strahler order (4–11) and calculated the mean values of diameters and lengths for each group. There was a strong correlation between Kassab’s empirical model and the constructed synthetic network diameters and lengths (Figure 3c,d).

Using a mixed-effects model (*n* = 679,205 observations), we quantified the diameter variability across the seeds and compared it to the empirical values. There was a strong positive relationship between the synthetic and empirical diameters (*p* < 0.001, standard error of 48 μm). The variance due to subjects and seeds was negligible (<0.001). These results suggest that the synthetic networks can accurately represent the physical properties of the arterial diameters, and the relationship between synthetic and empirical diameters is consistent regardless of the subject or seed.

**Figure 4:**
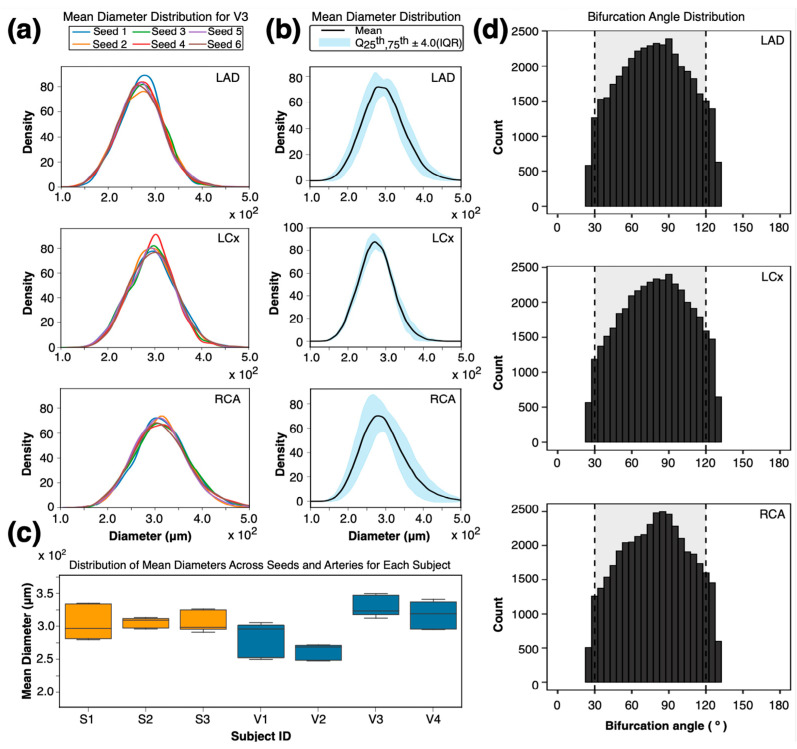
Diameters of terminal arterial segment across initial seeds and multiple subjects. (**a**) Variation in terminal diameter across various seeds for volunteer V3. (**b**) Terminal diameters for LAD, LCx, and RCA across multiple seeds and subjects. (**c**) Summary of mean terminal diameters across multiple arteries and seeds for each subject. (**d**) Histograms of bifurcation angles for LAD, LCx, and RCA across all subjects and seeds. Gray shading shows the physiological range (30–120°) reported in coronary morphometry studies. The mean ± SD angles were 78.0 ± 27.6° (LAD), 79.1 ± 27.5° (LCx), and 78.7 ± 27.4° (RCA).

The overall distributions of the terminal diameters for the LAD, LCx, and RCA across all seeds and subjects are shown in Figure 4. A sample distribution of the terminal diameter density in the three main coronary arteries of human volunteer V3 is shown in Figure 4a. Using a mixed-effect model (*n* = 252,000 observations), where the seeds were considered as the fixed effect while the subject and artery were considered as random effects, we showed an insignificant variability across various seeds with a standard error of 10 μm (*p*-values range from 0.12 to 0.98, Figure 4b), confirming that random initialization does not introduce a bias into generated diameters. The variance decomposition indicated that 97% of the explained variance arose from artery-level differences within subjects. Pairwise Kolmogorov–Smirnov comparisons between seeds stratified by artery yielded mean D-statistics of 0.0125 [0.0108–0.0141] for the LAD, 0.0125 [0.0107–0.0143] for the LCx, and 0.0110 [0.0095–0.0124] for the RCA (all *p* > 0.1), indicating that the terminal diameter distributions are statistically similar across seeds. Additionally, there was low variability in the mean terminal diameters across all seeds for each subject (Figure 4c), supporting consistency across different seeds and subjects. The lack of a significant difference between the seeds suggests that the framework is not dependent on the initial seed location, and the randomness of the seeds did not introduce significant variability in the diameters. The minimal subject-specific variability further supports the robustness and consistency of the framework’s output and stability based on in vivo, subject-specific data across two species.

To evaluate the branching geometry, we analyzed bifurcation angles across all generated arterial networks (Figure 4d). Each histogram represents the distribution of bifurcation angles across all subjects and seeds, with the physiological range of 30–120° [21,22,23] shaded in gray. The mean bifurcation angles were 78.0 ± 27.6° for the LAD, 79.1 ± 27.5° for the LCx, and 78.7 ± 27.4° for the RCA, with more than 90% of all bifurcations falling within the physiological range. The mixed-effects model indicated minimal differences between bifurcation angles across main epicardial coronary artery trees (<1°) despite the statistical significance due to the large sample size (*p* < 10^−7^). The effect of random seedings was negligible, confirming high reproducibility across stochastic initializations.

### 3.3. Hemodynamic Analysis of Subject-Specific Arterial Networks

**Figure 5:**
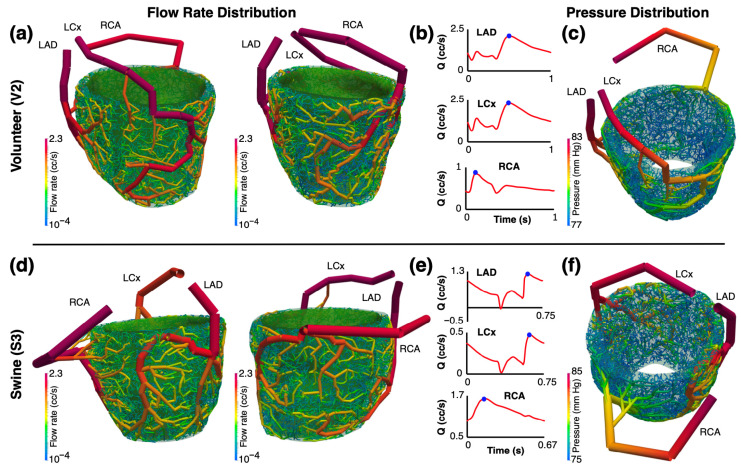
Blood flow rate and pressure across the entire arterial network. Video S2 shows a dynamic illustration of blood flow rate, and Video S3 shows pressure for human subject V1. Snapshots of (**a**) flow rate distribution in arterial networks, (**b**) blood flow waveforms, and (**c**) pressure distribution are shown for human subject V2 and swine (**d**–**f**). The blue dot (**b**,**d**) shows the onset of diastole, where the snapshots for the graphic depiction (**a**,**c**,**d**,**f**) were taken. Log scale was used for mapping data to colors for flow rate and pressure distribution.

To evaluate the functionality of the synthetic networks, the 1D pulsatile blood flow was simulated for all subjects and seeds (*n* = 126 simulations). Prior to each simulation, global optimization was performed to tune the diameters of each deformable arterial segment such that the mean standard error between the distribution of the blood flow rate at the terminal segments for each myocardial segment and FE-MRI fMBV-derived flow rates were less than $10^{-8}$. A general physiological flow rate waveform was prescribed at the root segment of each coronary arterial tree [13,14], and a linear elastic material model was considered for the arterial walls [15]. Figure 5 shows the flow rate and pressure distribution across the entire arterial network for volunteer V2 and swine S3. Snapshots were taken at the onset of diastole. Dynamic simulations are shown in the Supplementary Videos S2 and S3.

**Figure 6:**
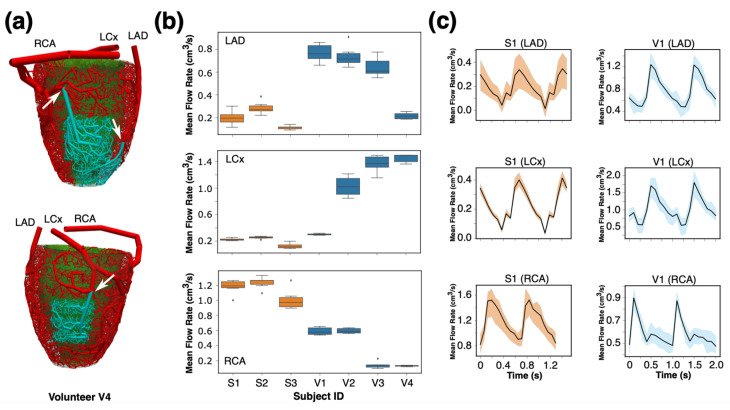
Hemodynamic analysis of synthetic arterial network across initializing seeds, arteries, and subjects. (**a**) Schematic representation of parent arteries supplying blood to the mid-inferoseptum, mid-anterolateral, and apical septum. The arrows point to the parent arterial segment where the flow rate waveforms were measured. (**b**) Variation in mean blood flow rate at target arterial segments for human (V1–V4) and swine (S1–S3) subjects (*n* = 126 simulations). Mixed-effects model shows low variability across seeds (*p* = 0.100 to 0.330). (**c**) Sample blood flow rate waveforms at the target segment supplying blood to the selected myocardial segment for subjects S1 and V1. Mixed-effects model shows minimal variability across seeds (*p* > 0.260, maximum standard error of 0.108 across three arteries). Shaded areas along the flow waveforms represent variation in flow rate across the initializing seeds.

To compare the flow rate and resistance across six seeds, the parent artery perfusing the mid-inferoseptum, mid-anterolateral, and apical septum was selected (Figure 6). A mixed-effects model (*n* = 126 observations) was applied to evaluate the variability of the synthetic mean flow rate across the seeds and subjects. We found minimal variability in the mean blood flow rate across seeds (*p*-values = 0.039 to 0.33; fixed effect: seeds; and random effect: artery, subject, and subject type). For seed 3, the effect was minimally significant (*p* = 0.039 with a coefficient of 0.048 cc/s). There was moderate variability across subjects and arteries (LCX [*n* = 42 observation, variance = 0.106] > RCA [*n* = 42 observation, variance = 0.092] > LAD [*n* = 42 observation, variance = 0.055]). The mixed-effect model demonstrated non-significant variability in the calculated resistances across seeds at the target segments (*p* = 0.21 to 0.99). The group variance in all three arterial segments was larger than the seed variance. These findings suggest that the variability in resistance was primarily attributed to differences between subjects rather than between seeds and highlight within-subject consistency.

We further evaluated the variability of the flow rate waveforms in the parent artery supplying blood to aforementioned myocardial segments using the DTW distance as a measure of waveform dissimilarity [19]. Mixed-effects models (*n* = 105 observation for each epicardial coronary artery group) were applied to determine if the variability in DTW distances was significantly influenced by different subjects and seeds for each main coronary arterial tree. DTW distances between blood flow rate waveforms from different seeds for each subject were calculated, and a mixed-effects model was used to quantify the effect of subjects and seeds on the DTW distances. For the LAD, the choice of the seed location did not significantly affect the flow rate waveforms (*p* = 0.26 to 0.78). The maximum standard error was 0.11 cm^3^/s across three arteries. Notable variability was observed between subjects, as indicated by the subject variance in all three arteries (0.042, 0.082, and 0.076 for the LAD, LCx, and RCA, respectively). Our model not only captured the inherent variability in the flow rate waveforms between subjects but also maintained within-subject consistency when various seeds were used to construct the microvascular networks. Further, the difference in the initial seed location did not significantly impact the within-subject blood flow waveform at different sections of the coronary arterial network. The consistency across the seeds underscores the reliability and repeatability of our microvascular network generation procedure for predicting the coronary artery flow rates at distal arterial segments.

### 3.4. Verification of In Silico Fractional Myocardial Blood Volume Maps from Synthetic Arterial Networks

**Figure 7:**
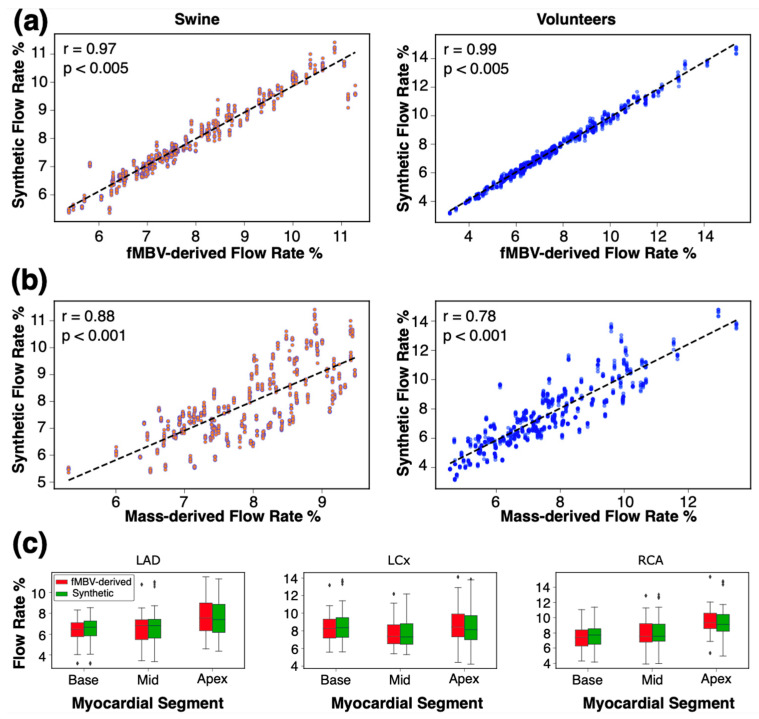
Replication of FE-MRI-derived and mass-derived fMBV maps by synthetic microvascular networks. The data reflect *n* = 960 observations in four volunteers and *n* = 684 observations in three swine. (**a**) Comparison of segmental blood flow rate percentages (SBFPs) from synthetic arterial networks vs. FE-MRI fMBV-derived SBFPs. (**b**) Comparison of SBFPs from synthetic vs. mass-derived SBFPs. (**c**) Median and interquartile ranges for SBFP derived from synthetic networks and FE-MRI fMBV maps.

To assess the performance of our framework to replicate the fMBV maps, we compared the SBFPs derived from our synthetic arterial network with FE-MRI-derived and mass-derived SBFPs. Figure 7 shows the aggregated synthetic SBFP across all subjects, myocardial segments, and seeds against the fMBV-derived SBFP and mass-derived SBFP. There is minimal variability in synthetic SBFPs and a strong correlation with internal reference values.

**Figure 8:**
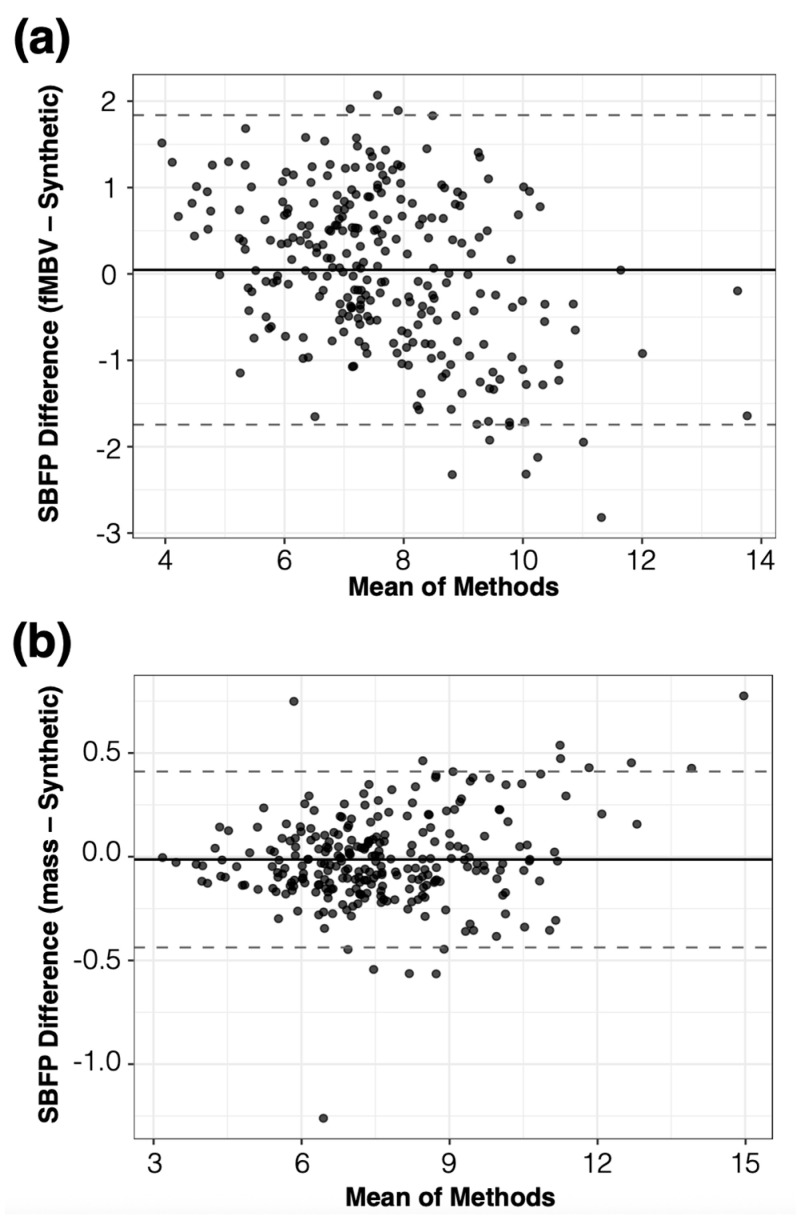
Agreement between synthetic and reference segmental blood flow percentage (SBFP) values. Bland–Altman plots comparing synthetic SBFP to fMBV-derived SBFP (**a**) and myocardial-mass-derived SBFP (**b**) across all subjects. Solid lines indicate mean bias; dashed lines represent ±1.96 SD limits of agreement.

To quantify the variation in SBFPs across the seeds and their correlation with the reference fMBV- and mass-derived SBFP while considering the effect of subjects and the myocardial segment location (basal, mid, and apical) and arterial territories, we used mixed-effect models. The Bland–Altman comparison across all subjects and myocardial segments is presented in Figure 8. For volunteers (*n* = 960 observations), the synthetic SBFP was lower than the reference with an intercept of −0.065% (*p* = 0.048), without a significant difference across various seeds (*p* > 0.99). A significant positive correlation was found between the synthetic SBFP and the fMBV-derived percentages, with a coefficient of 0.99 (*p* < 0.005). In human volunteers, the synthetic SBFP closely matched fMBV-derived distributions (RMSE = 0.22%) with high replicability (ICC = 0.99, 95% CI 0.99–0.99). The agreement with the mass-derived SBFP was moderate but physiologically consistent (RMSE = 0.91%, ICC = 0.85, 95% CI 0.81–0.88). For swine (*n* = 684 observations), the synthetic SBFP was higher than the reference by 0.347% (*p* < 0.001; ICC > 0.97), without significant subject- and myocardial segment-related effects. These results show a strong correspondence between the output of our synthetic microvascular network and the MRI-fMBV-derived SBFP. The lack of significant variability across initializing seeds supports the reliability of our framework.

To evaluate the robustness of the fMBV-derived flow constraints, we performed a steady-state sensitivity analysis in the LAD arterial network of subject V3 (Seed 1). Four key parameters, including blood viscosity, wall stiffness in the Olufsen arterial wall model, distal terminal pressure, and the inlet flow rate, were independently perturbed from their baseline values. The resulting changes in the SBFP were quantified using the root-mean-square deviation relative to baseline distributions. As summarized in Table 1, synthetic SBFP values remained stable across all perturbations, confirming that the territorial flow allocation is robust to uncertainties in rheological and boundary parameters. These findings demonstrate that the synthetic networks generated using the fMBV-derived SBFP preserve blood flow distribution patterns despite variations in model inputs.

**Figure 9:**
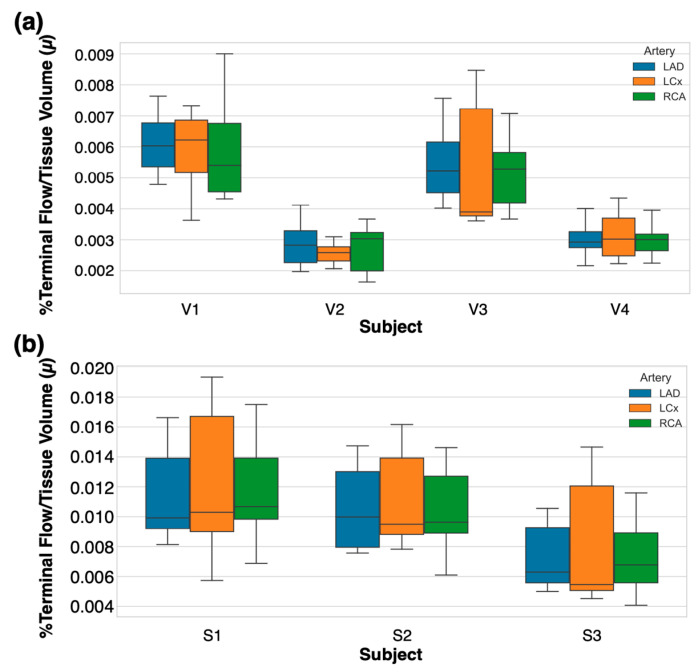
Terminal vascular-tissue flow delivery *μ* across arterial territories. Boxplots show *μ* for LAD, LCx, and RCA in (**a**) healthy volunteers and (**b**) swine. Horizontal lines indicate medians; whiskers denote 1.5 × IQR. Mixed-effects modeling revealed no significant fixed effect of arterial territory (*p* > 0.05), confirming consistent terminal distribution per tissue volume across arteries and subjects. Between-subject variability (ICC = 0.62) dominated over artery effects, reflecting physiological inter-subject variations in *μ*.

To assess the physiologic proportionality between the terminal density and perfused tissue volume, we computed the terminal vascular-tissue flow delivery μ for all healthy volunteers and swine subjects. As shown in Figure 9, the value of μ was consistent across the LAD, LCx, and RCA territories for both healthy volunteers and swine, with overlapping interquartile ranges. Mixed-effects modeling (random intercepts for subject and seeds; number of observations = 672) confirmed the absence of significant effects for the artery (*p* = 0.08 for LCx, *p* = 0.98 for RCA), indicating uniform terminal vascular-tissue flow delivery across major coronary territories.

The ICC demonstrated that approximately 62% of the variance is because of differences between subjects in myocardial volume and perfusion patterns, while the contributions from the artery and seeding were negligible. These findings confirm that the arterial network generation algorithm preserves physiologically realistic territory scaling, such that the terminal segment density aligns with the perfused myocardial tissue volume independently of the artery or cohort.

### 3.5. Arterial Network Generation for a Patient with Ischemic Heart Disease

**Figure 10:**
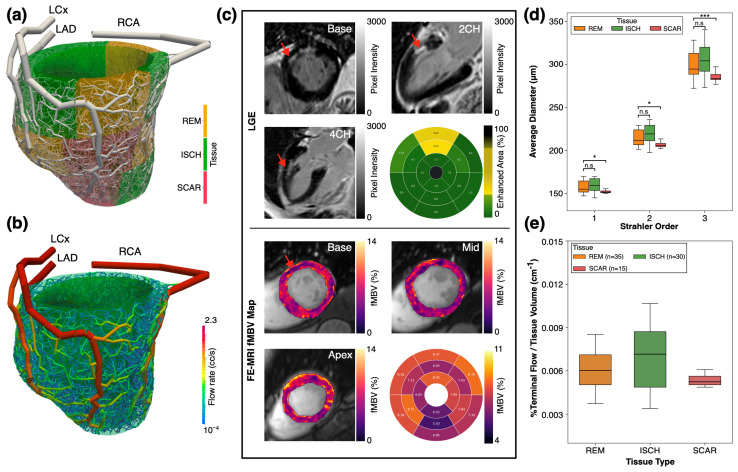
Microvascular network constructed for a patient with ischemic heart disease (IHD) showing inhomogeneous morphology and flow. (**a**) A 3D representation of the constructed microvascular networks and labeled myocardial segments. (**b**) Snapshot of flow rate distribution in the arterial tree at the onset of diastole. (**c**) Examples of late gadolinium enhancement (LGE) MRI with scar tissues and ferumoxytol-enhanced MRI fractional myocardial blood volume (FE-MRI fMBV) maps. The arrows point at tissues with scar. The accompanying polar maps quantify regions of remote, ischemic, and scar tissues. (**d**) The mean arterial segment diameter for Strahler order numbers 1–3, categorized by each tissue type. (**e**) Variation in the ratio of total coronary flow percentage and tissue volume for each terminal segment in each tissue type. REM: Remote. ISCH: Ischemia. Asterisks indicate statistical significance: * *p* < 0.05, *** *p* < 0.001, and n.s. *p* > 0.05.

Synthetic arterial network, MRI data, and analysis of coronary morphometry and hemodynamics for an IHD patient are shown in Figure 10.

The mean arterial segment diameters for Strahler order numbers 1, 2, and 3, categorized by each tissue type, are shown in Figure 10d. Across the considered Strahler order range of 1–3, tissues with a reversible perfusion defect (ischemia) showed the largest average arterial segment diameters (average diameter for Strahler order number 1: 160 ± 8 μm, *n* = 30), while the tissue with a fixed perfusion defect and LGE (scar) showed smaller average arterial segment diameters (average diameter for Strahler order number 1: 152 ± 2 μm, *n* = 15). Mann–Whitney U tests found statistically significant differences between the diameters of arterial segments in remote and scar tissues across the considered Strahler order range (Benjamini–Hochberg adjusted *p* < 0.05). These differences were the most prominent at Strahler order number 3, where vessel diameters are larger, and, as a result, the difference in the hemodynamic environment is more pronounced (remote–scar: 95% bootstrap CI [9.47, 20.52], *p* < 0.001; remote–ischemic: 95% CI [−12.75, 2.19], *p* = 0.14). As expected, the terminal vascular-tissue flow delivery μ at rest for scar tissue was lower than remote and ischemic tissues (scar 0.0053 ± 0.0005 cm^−3^, *n* = 15; remote 0.0060 ± 0.0013 cm^−3^; *n* = 35; ischemic 0.0069 ± 0.0022 cm^−3^; and *n* = 30). Notably, μ was higher for all three tissue types in this patient with three-vessel coronary disease and scars when compared to the healthy myocardial tissue of the volunteers (0.0039 ± 0.0014 cm^−3^; *n* = 144), which is consistent with chronic three-vessel coronary disease of varying severities that induce collateralization. This illustrative example provides additional evidence suggesting that both geometric (diameter) and functional (μ) signatures align with the expected autoregulatory behavior where the arteriolar dilation and capillary recruitment of the microcirculation are observed in both ischemia and non-transmural scars.

## 4. Discussion

This proof-of-concept study explores the feasibility of constructing subject-specific synthetic coronary arterial networks from ferumoxytol-enhanced coronary MRA and MRI fMBV maps. By coupling an adaptive, multistage CCO algorithm with fMBV maps, the framework aims to represent the microvascular anatomy and autoregulatory behavior using a physiologically constrained, image-based pipeline. Our findings in healthy swine, human volunteers, and a patient with ischemic heart disease support the potential of the proposed framework to non-invasively compute key coronary hemodynamic measures.

Despite an exponential increase in the cost burden [24], the management of chronic IHD focuses on obstructive epicardial coronary arteries as the primary cause of angina [25]. However, three out of four patients with angina undergoing invasive angiography do not show signs of epicardial coronary obstruction [26]. Instead, coronary microvascular dysfunction (CMD) is the main contributor. In women, CMD results in a five-fold increase in major adverse cardiovascular events [25,27]. Current strategies to definitively diagnose symptomatic CMD rely on an invasive coronary evaluation, which includes (1) the exclusion of significant anatomic obstructive epicardial coronary disease, (2) provocative vasoreactivity testing, and (3) quantification of the index of microcirculatory resistance (IMR) [25].

The findings from this work support the use of patient-specific MRI data to non-invasively evaluate microvascular health through the synthetic arterial network generation and non-invasive computation of coronary hemodynamics. The distinguishing feature of our approach relies on the incorporation of FE-MRI fMBV maps into microvascular network construction. When used in combination with a two-compartment water exchange model, the fMBV maps account for the underlying autoregulatory mechanisms and provide accurate representations of the ferumoxytol distribution as a surrogate for the intravascular blood volume—unlike gadolinium-based contrast agents in MRI or iodine-based CT contrast agents that immediately leak into the extravascular space. At a concentration of 4 mg/kg or less, the relationship between the ferumoxytol concentration and MRI signal is also linear, which differs from many nuclear tracers that have nonlinear relationships. These properties provide a physiological surrogate for the intravascular blood volume and are used to encode aspects of autoregulation in the microvascular network construction.

Recent computational efforts have used a Darcy model to approximate the microcirculation while integrating CT-MPI with truncated synthetic coronary tree generation models to reproduce the myocardial blood flow estimates [8,28]. Although porous media models are effective in specific applications, they oversimplify the anatomic branching and flow directionality. On the other hand, CT-MPI has limitations related to the leakage of the contrast and the misalignment between acquisitions used to capture the coronary tree vs. perfusion information. Our synthetic framework directly generates the microvascular network and extends the epicardial coronaries based on the distribution of FE-MRI fMBV maps. Unlike the majority of CCO-based methods used to generate arterial trees [7,29,30], the arterial networks generated by our proposed algorithm are not binary, and the synthetically generated arterial segments represented the extension of previous terminal segments. The terminal segment flow rate across the AHA’s 16 myocardial segments is also different and represents fMBV map values. Additionally, this framework is capable of scaling the network resolution to 6000 terminal segments in the myocardium, embedding the autoregulatory aspects into the structure of the distal vasculature. Hence, it provides a more reliable surrogate for the complete arterial network to synthetically estimate the hemodynamic environment of a patient-specific LV using flow waveforms and resistances. Compared to empirical swine models, our preliminary results showed high correlation coefficients while maintaining minimal variability across various random seedings in healthy subjects. In a subject with mixed myocardial perfusion defects, our framework reconstructed heterogeneous microvascular networks with an increased number of terminal vessels in the myocardial segment with reversible perfusion defects while distributing a less dense microvascular tree in segments with fixed perfusion defects. These results indicate that the proposed adaptive CCO approach may address the oversimplification of Darcy-based models of the microcirculation while observing anatomic accuracy and blood flow distributions, which are needed for modeling the key coronary hemodynamic measures such as the fractional flow reserve (FFR), IMR, and coronary flow reserve (CFR).

Recent machine learning surrogates have demonstrated a remarkable capability to estimate coronary hemodynamics directly from anatomical data, including geometry-based predictions of the FFR and wall shear stress distributions in near real time [31,32,33]. These models achieve a rapid inference suitable for high-throughput triages but are typically limited to the epicardial coronary level and do not explicitly encode tissue-specific microvascular density, autoregulatory mechanisms, or transmural loading conditions that govern the myocardial perfusion reserve. In contrast, the proposed FE-MRI fMBV-based, physiology-constrained framework integrates quantitative microvascular information derived from ferumoxytol-enhanced MRI into a mechanistic arterial network model, enabling the simulation of flow distributions and resistances. While machine learning surrogates emphasize speed and data scalability, the present framework prioritizes physiological interpretability and the ability to compute functional indices (e.g., CFR, IMR, and FFR) within a patient-specific arterial network. Together, these approaches are complementary, such that machine learning algorithms can accelerate initial geometric screening and provide boundary conditions, whereas the proposed framework captures the multiscale hemodynamic environment underlying microvascular dysfunction. The combination of these methods, leveraging data-driven surrogates for efficiency and physiology-based modeling for interpretability, represents a promising direction for translational coronary hemodynamics. Future hybrid approaches that combine physiology-based vascular generation with learned surrogates for flow or boundary condition estimation may enable physiologically accurate, real-time predictions of complete coronary arterial networks, bridging the current gap between data-driven and mechanistic methodologies.

Our findings in the patient with IHD demonstrate the potential of the proposed framework to reflect the autoregulation mechanism in the microvascular bed captured through the use of fMBV maps. In the IHD subject with mixed ischemia and scar tissues, the microvascular networks generated across five random seedings showed a tissue-dependent geometry and vascular-tissue flow delivery (μ), consistent with coronary autoregulation [34,35]. In tissues with ischemia, the average terminal arterial segments were larger than the terminal tree in the remote tissue. This behavior is expected under reduced perfusion pressure, where the microvascular dilation lowers the distal resistance and compensates for the segmental flow at rest, increasing the ability of the microvasculature to deliver blood to the tissue [35]. On the other hand, smaller terminal diameters were observed in the tissue with scar (fixed perfusion defect and late gadolinium enhancement) and hence were related to the lower terminal flow delivery capability. This behavior is consistent with the microvascular rarefaction and increased extravascular resistance in fibrotic tissue [36,37].

### 4.1. Limitations

Our study has several limitations. This study was conducted in a small cohort, and the findings should be interpreted as preliminary. However, we show the potential of the framework in idealized myocardium, healthy swine, human subjects, and one patient with mixed myocardial perfusion defects. Arterial segments were assumed to be cylindrical without explicit tapering to reduce the computational cost. Although tapering is implicitly represented via Murray’s law at bifurcations, continuous axial tapering was not imposed. Incorporating smooth taper functions will further improve the morphological realism. The synthetic arterial network morphometry was validated against empirical swine models, which may not fully capture the complexity of the swine coronary circulation, specifically in diseased myocardium. Population-averaged coronary waveforms and the constant outlet pressure were used as inflow and outflow boundary conditions, respectively. While this approach is common in coronary modeling, it neglects subject-specific variations in the heart rate and distal impedance. A detailed microvascular rheology, including the Fåhræus–Lindqvist viscosity model and red cell phase separation at bifurcations, was not modeled. These effects primarily influence vessels < 100 μm [38] and are secondary to the macro-scale flow constraints considered in this study. Sensitivity analyses with varying viscosities and wall stiffnesses across physiological ranges produced negligible changes in the SBFP, suggesting a limited impact on the hemodynamic environment. The present analysis focuses on steady-state perfusion at rest and does not model time-dependent myocardial deformation or cyclic intramyocardial pressure. This simplification is justified because the coronary flow occurs predominantly during diastole, when the myocardial contraction is minimal. The ferumoxytol-enhanced MRI data used as input were acquired under steady-state, end-diastolic conditions and therefore already reflect the net effects of the wall stress on the microvascular filling. While the framework showed low variability across various seeds and subjects, the influence of epicardial coronary artery disease as well as microcirculatory diseases (such as microvascular spasm) on our synthetic arterial networks was not investigated. The computational cost of the microvascular network construction and hemodynamic simulation in large networks needs to be reduced. The end-to-end pipeline requires approximately two days per subject on a single CPU core (see Supplementary Material S3), with arterial network generation as the principal bottleneck. The parallelization and optimization of the framework performance and improving its efficiency will be necessary for clinical applications. The fMBV values were quantified from six to eight left ventricular short axis images with a slice thickness of 8 mm and an interpolated spatial resolution of 0.93 × 0.93 mm^2^, which may limit the volumetric precision.

Despite the aforementioned limitations, the proposed framework generated a consistent network morphometry and hemodynamic environment across healthy and IHD subjects and initial seedings, while conforming to patient-specific left ventricular anatomy and flow boundary conditions. These findings demonstrate potential for using the fMBV to generate synthetic microvascular networks, which could provide a powerful non-invasive tool for estimating key hemodynamic indices.

### 4.2. Future Work

Future studies could extend the current validation to patients with ischemic and microvascular disease. In these cohorts, PET- or CT-derived perfusion indices and invasive measurements such as the FFR and IMR will provide quantitative benchmarks. This will test how disease-related alterations in vessel density and epicardial coronary artery disease affect the morphology and flow distribution. While the present study uses steady-state fMBV maps to derive SBFPs, future work could integrate conventional flow-based indices from dynamic perfusion imaging modalities to directly compare perfusion-derived and fMBV-derived metrics within the same framework, thereby bridging intravascular volume and flow-based physiological assessments. Additionally, incorporating non-Newtonian viscosity models will enable a more realistic simulation of the microvascular rheology. These additions will improve accuracy for vessels under 100 µm in diameter. The long-term goal is to achieve a fully automated, physiology-aware framework capable of the same-day computation of perfusion and functional indices from standard perfusion imaging modalities. Integration with machine learning surrogates may accelerate the initial network generation, while the mechanistic model will retain physiological interpretability and support personalized risk assessments.

## 5. Conclusions

We introduced an innovative framework for generating synthetic arterial networks based on FE-MRI fMBV maps. This approach is capable of estimating subject-specific coronary blood flow profiles that account for microcirculatory autoregulatory mechanisms. Although further research is required to optimize and validate the model in patients with pathological conditions, the early findings support its potential to non-invasively quantify critical hemodynamic parameters. Furthermore, its potential compatibility with other perfusion imaging modalities enhances its promise as a valuable tool.

## 6. Patents

Kim-Lien Nguyen, Mostafa Mahmoudi, and Amirhossein Arzani are co-inventors on a provisional patent application entitled “Systems and methods for adaptive synthetic microvascular network generation” (U.S. Provisional Patent Application No. 63/659,295), which pertains to the methodology described in this manuscript.

## Figures and Tables

**Table 1:** Sensitivity of segmental blood flow percentage (SBFP) to physiological parameter perturbations in the LAD arterial network of subject V3 (Seed 1). Each parameter was varied independently around its baseline value under steady-state conditions. Values are reported as mean ± standard deviation across all myocardial segments and variations. CoV, coefficient of variation; RMSE, root-mean-square error.

Parameter	Variation	CoV (%)	RSME (%)
Viscosity	−16–21%	0.0048 ± 0.0003	0.0006 ± 0.0004
Stiffness	−20–20%	0.0560 ± 0.0037	0.0049 ± 0.0020
Inflow Rate	−20–20%	0.0021 ± 0.0001	0.0002 ± 0.00007
Terminal Pressure	−20–20%	0.1597 ± 0.0106	0.0127 ± 0.00491

## Data Availability

The data and computational methods supporting the findings of this study are available from the corresponding author upon reasonable request. Access to the imaging datasets and computational code requires the execution of a Material Transfer Agreement (MTA) and/or Data Use Agreement (DUA) with the authors’ institution.

## Supplementary Materials

The following supporting information can be downloaded at https://www.mdpi.com/article/10.3390/bioengineering12111274/s1: S1. Legend for Supplementary Videos; S2. Parameters and Model Specification; S3. Pipeline Runtime; S4. Morphometric Validation; S5. Results from Statistical Analysis; and S6. References in Supplemental Methods. References [9,13,14,15,17,19,21,22,23,38,39,40,41,42] are cited in Supplementary Materials.
