# Supplementary material for: Multiscale Coronary Arterial Network Generation and Hemodynamics Using Patient-Specific Fractional Myocardial Blood Volume
Source: Bioengineering (Basel). 2025 Nov 20;12(11):1274. doi: 10.3390/bioengineering12111274 (PMC12649997; doi:10.3390/bioengineering12111274)
Supplement: Supplementary file 1 [file bioengineering-12-01274-s001.zip › bioengineering-3926531-supplementary.pdf]

SUPPLEMENTAL MATERIAL

to

Multiscale Coronary Arterial Network Generation and Hemodynamics Using Patient-Specific Fractional Myocardial Blood Volume

Mostafa Mahmoudi, Ph.D.[1,2], Arutyun Pogosyan, M.D.[1], Amirhossein Arzani, Ph.D.[3,4], Kim-Lien Nguyen, M.D.[1,2,5]

[1] Division of Cardiology, David Geffen School of Medicine at UCLA and VA Greater Los Angeles Healthcare System, Los Angeles, CA, USA
[2] Department of Bioengineering, University of California Los Angeles, Los Angeles, CA, USA
[3] Department of Mechanical Engineering, University of Utah, Salt Lake City, Utah, USA
[4] Scientific Computing and Imaging Institute, University of Utah, Salt Lake City, Utah, USA
[5] Department of Radiological Sciences, David Geffen School of Medicine at UCLA, Los Angeles, CA, USA

List of Supplemental Sections:

S1. Videos
S2. Parameters and Model Specification
S3. Pipeline Runtime
S4. Morphometric Validation
S5. Results from Statistical Analysis
S6. References in Supplemental Methods

S1. Supplementary Videos

Supplementary Video S1. Timelapse of coronary microvascular network generation for human volunteer V1. The total number of generated arterial segments was 16111, with 6000 terminal segments (radius of 130.2 ± 38.9 microns). The animation illustrates the step-by-step generation of the microvascular tree, focusing on (a) the left anterior descending (LAD), (b) left circumflex (LCx), (c) right coronary artery (RCA), and (d) the complete coronary arterial network.

Supplementary Video S2. Timelapse of blood flow rate (cm^3/s) distribution across the entire coronary arterial network (LAD, LCx, RCA) of human volunteer V1 (comprising n=16,111 arterial segments). The animation demonstrates the blood flow distribution over the course of ten consecutive heartbeats.

Supplementary Video S3. Timelapse of blood pressure (mmHg) within the entire coronary arterial network (LAD, LCx, RCA) of human volunteer V1 (comprising 16,111 arterial segments). The animation displays the pressure distribution across 10 consecutive cardiac cycles.

S2. Parameters and Model Specification

All one-dimensional (1D) hemodynamic simulations and synthetic arterial network generation steps were implemented using parameter set and boundary conditions (BC) derived from physiological and literature-based data. The epicardial coronary arteries, left ventricular myocardium, and fMBV values were extracted from MRI. The purpose of this section is to provide comprehensive transparency regarding the assumptions, constants, and solver configurations employed in both the proposed microvascular network generation framework and hemodynamic simulations.

TABLE S1
PHYSIOLOGICAL AND BOUNDARY PARAMETERS USED IN ARTERIAL NETWORK GENERATION AND HEMODYNAMIC SIMULATIONS.

**Parameter**	**Value**	**Units**	**References**
Inflow BC (1D Hemodynamic)	Waveform at proximal LAD, LCx, RCA	[cc/s]	[1,2]
Inflow BC (CCO)	LAD: 0.48<br>LCx: 0.47<br>RCA: 0.64	[cc/s]	[3]
Outflow BC (1D hemodynamic)	77	[mmHg]	-
Outflow BC (CCO)	Perf. pressure = 80	[mmHg]	[4]
Terminal count per Subject	6000	-	-
Material- stiffness constant	1.5e4	[mmHg]	[5]
Material- exponential constant	-25.5	[1/cm]	[5]
Material- offset constant	349	[mmHg]	[5]
Blood viscosity	3.6	[cP]	[6]pr
Minimum L/D ratio	2	[-]	-
Bifurcation angle constraint	25-240	[degrees]	[7–9]
Bifurcation symmetry ratio	0.1 – 0.7	[-]	[4]

TABLE S2
NUMERICAL SOLVER CONFIGURATION AND HARDWARE ENVIRONMENT

	Parameter	Value
1D Solver	Time step	0.002 s
	Total cardiac cycle	10
	Convergence criteria	<1e-6
	Iterative scheme	Second order
Simulation environment	Programing language	C++14
	OS	iOS/CentOS
	CPU	Intel/M1

S3. Pipeline Runtime and Computational Performance

TABLE S3
STAGE-WISE RUNTIME AND OPERATOR INVOLVEMENT FOR EACH SUBJECT

Stage	Description	Runtime estimate	Output type	Notes
1	Myocardial and coronary segmentation in 3D Slicer	1 hr	STL and VTU files	Manual operation
2	Image registration and fMBV mapping quantification	0.3 hrs	DICOM images	Automated (minor manual ROI)
3	fMBV segmentation	0.3 hrs	TXT files	Manual operation
4	Adaptive, multi-stage, CCO arterial network generation	24 hrs	TXT and VTK files	Automated
5	Global diameter optimization	4 hrs	TXT and VTK files	Automated
6	1D hemodynamic simulation for 10 cardiac cycles	5 hrs	TXT and VTK files	Automated
7	Post-processing	0.4 hrs	TXT and VTK files	Manually executing codes
	Total per-subject:	35 hrs		

The Adaptive, multi-stage, CCO arterial network generation algorithm uses 1 CPU core with 20GB memory. Global diameter optimization and 1D hemodynamic simulation run on 8 CPU cores and 20 GB memory.

S4. Morphometric Validation

Quantitative morphometric analyses were conducted to assess the anatomical similarity of the synthetic arterial networks relative to established empirical morphometry. All comparisons were made against the porcine coronary dataset of Kassab et al. [10], which provides order-specific means and standard deviations for segment diameters and lengths. The synthetic networks generated for seven subjects (six seeds per artery) were pooled within each major coronary territory (LAD, LCx, RCA). Deming regression was used to account for measurement uncertainty in both morphological parameters, and intraclass correlation coefficients (ICC, 95 % CI) quantified agreement strength of agreement. Root-mean-square error (RMSE) and mean absolute percentage error (MAPE) were computed to summarize the absolute deviations. The analyses were stratified by artery (LAD, LCx, RCA) and descriptor (segment diameter, segment length).

TABLE S4

DEMING REGRESSION AND AGREEMENT METRICS VERSUS KASSAB'S EMPIRICAL MODEL: HUMAN SUBJECTS. DIAMETERS ARE PRESENTED IN [μ] AND LENGTHS ARE IN [MM] UNITS

Artery	Metric	Slope [95% CI]	Intercept [95% CI]	ICC [95% CI]	RSME	MAPE
LAD	Diameter	1.05 [0.78, 1.31]	-502 [-917, -87]	0.97 [0.85, 0.99]	506	230
LAD	Length	2.08 [1.35, 2.80]	0.13 [-0.37, 0.62]	0.75 [0.18, 0.95]	0.85	66
LCx	Diameter	0.99 [0.70, 1.29]	-454 [-829, -79]	0.95 [0.79, 0.99]	527	635
LCx	Length	2.80 [1.45, 4.14]	-0.03 [-0.96, 0.90]	0.60 [-0.05, 0.89]	1.2	90
RCA	Diameter	0.91 [0.68, 1.14]	-470 [-855, –85]	0.96 [0.83, 0.99]	655	442
RCA	Length	0.79 [0.44, 1.14]	0.51 [0.01, 1.00]	0.88 [0.55, 0.97]	0.62	66

Diameter regressions show slopes near 1 and high ICCs (> 0.94), confirming strong geometric fidelity. Length relations exhibit greater spread but remain within 12% acceptance band of Kassab's empirical model.

TABLE S5

DEMING REGRESSION AND AGREEMENT METRICS VERSUS KASSAB'S EMPIRICAL MODEL: SWINE SUBJECTS. DIAMETERS ARE PRESENTED IN [μM] AND LENGTHS ARE IN [MM] UNITS

**Artery**	**Metric**	**Slope [95% CI]**	**Intercept [95% CI]**	**ICC [95% CI]**	**RSME**	**MAPE**
LAD	Diameter	0.95 [0.72, 1.18]	-458 [-825, -92]	0.96 [0.85, 0.99]	587	394
LAD	Length	2.84 [1.73, 3.94]	-0.01 [-0.58, 0.56]	0.61 [-0.04, 0.90]	0.93	72
LCx	Diameter	0.87 [0.57, 1.16]	-482 [–940, –25]	0.94 [0.72, 0.99]	720	505
LCx	Length	2.47 [0.65, 4.29]	0.29 [-1.08, 1.66]	0.62 [-0.08, 0.91]	1.31	73
RCA	Diameter	1.06 [0.83, 1.30]	-475 [-815, -135]	0.97 [0.87, 0.99]	472	359
RCA	Length	1.39 [0.72, 2.06]	0.44 [-0.15, 1.02]	0.84 [0.45, 0.96]	0.79	66

Swine networks mirror empirical morphometry with slopes close to 1 for diameter and > 1 for length, consistent with mild over-branching at small orders. All diameter ICCs > 0.93 confirm high reproducibility.

S5. Statistical analysis

Mixed-effects models were employed to analyze the variability in arterial diameters, flow rates, and flow resistance across various seeds, main coronary arteries, and subjects. These models account for both fixed effects (seeds) and random effects (coronary arteries, myocardial segments, and subjects), providing an analysis of the factors influencing the observed variability. Dynamic Time Warping (DTW)[11] was employed to measure the dissimilarity between flow rate waveforms from different seeds for each subject and artery. This technique aligns flow rate waveforms to minimize the distance between them, enabling an accurate comparison of their shapes. In conjunction with the mixed-effect model, DTW was used to quantify the effects of subject, artery, and initial seeds on the variability in flow rate waveforms. This analysis was essential for assessing the repeatability of the hemodynamic results obtained from our synthetic framework. All statistical analyses were conducted using Python 3.9.16, with relevant libraries including SciPy[12], StatsModels[13], and DTW-Python[14].

Table S6 summarizes the Pearson correlation coefficients between the morphometry of generated arterial networks and the empirical models.

TABLE S6

AGREEMENT BETWEEN THE MORPHOMETRY OF SYNTHETIC ARTERIAL NETWORKS AND THE EMPIRICAL VALUES

**Subject type**	**Parameter**	**Pearson correlation coefficient**		
		**LAD**	**LCx**	**RCA**
Volunteers†	Diameter	0.99	0.98	0.99
	Length	0.95	0.80	0.92
Swine†	Diameter	0.99	0.99	0.99
	Length	0.87	0.92	0.79

†All p-values <0.001.

LAD, left anterior descending; LCx, left circumflex; RCA, right coronary artery

Table S7 presents the outcomes of the statistical analyses examining the correlation between empirical and synthetic diameters. The mixed-effects model assesses the relationship between empirical diameter (dependent variable) and synthetic diameter (fixed effect. The model incorporates random intercepts and slopes at the subject level, complemented by a variance component attributable to seeds. The fixed effect for diameter is highly significant (p<0.001), with an estimated coefficient of 933.2, signifying a strong positive correlation.

TABLE S7

MIXED-EFFECTS MODEL RESULTS FOR PREDICTING EMPIRICAL DIAMETERS (NUMBER OF OBSERVATIONS: 679,205).

**Parameter**	**Estimate**	**95% CI**	**p-value**
Empirical Diameter	-21.5 (intercept)	-29.6, -13.4	< 0.001
Synthetic Diameter	933.2	837.6, 1028.7	< 0.001

Table S8 presents the outcomes of the mixed-effects model for terminal artery diameters. The dependent variable analyzed is the observed terminal artery diameter. Seeds were incorporated as fixed effects, with subject and artery included as random effects. The intercept signifies the mean diameter across all seeds and arteries. The fixed effects for C(Seed) represent deviations from the reference seed (Seed 1). Variance components attributable to the artery were estimated as random effects. Statistical significance is denoted by P<0.05.

TABLE S8
MIXED-EFFECTS MODEL RESULTS FOR THE EFFECT OF VARIOUS SEEDING LOCATIONS ON THE TERMINAL ARTERY DIAMETER (NUMBER OF OBSERVATIONS: 252000).

Parameter	Estimate	95% CI	p-value
Seed 1	0.028 (intercept)	0.026, 0.029	< 0.001
Seed 2	$<10^{-4}$	~0, ~0	0.88
Seed 3	$<10^{-4}$	~0, ~0	0.24
Seed 4	$<10^{-4}$	~0, ~0	0.98
Seed 5	$<10^{-4}$	~0, ~0	0.12
Seed 6	$<10^{-4}$	~0, ~0	0.77

Table S9 presents the findings derived from the mixed-effects model examining mean flow rate measurements across all subjects and arteries. The results indicate that the baseline mean flow rate for Seed 1 is 0.570 (95% CI: 0.374–0.766). Among the seed locations, only Seed 3 demonstrates a small yet statistically significant increase in flow (p=0.039) in comparison to Seed 1. Seed 2 exhibits a marginally significant increase (0.045; p=0.051), whereas the remaining seeds show non-significant positive differences relative to Seed 1. This suggests minimal variability in mean flow rate among subjects, with the artery-level variance (0.204) accounting for the majority of the variability in mean flow rate.

TABLE S9
MIXED-EFFECTS MODEL RESULTS FOR MEAN FLOW RATE (NUMBER OF OBSERVATIONS: 126). FIXED EFFECTS CAPTURE THE DEVIATIONS FOR SEEDS (SEED 2–SEED 6).

Parameter	Estimate	95% CI	p-value
Seed 1	0.570 (intercept)	0.374, 0.766	< 0.001
Seed 2	0.045	-0.001, 0.091	0.051
Seed 3	0.048	0.002, 0.093	0.039
Seed 4	0.035	-0.011, 0.080	0.133
Seed 5	0.027	-0.018, 0.072	0.244
Seed 6	0.023	-0.023, 0.068	0.331

Table S10 presents the results of the mixed-effects model for blood flow resistance in the left anterior descending (LAD) artery. The intercept denotes the baseline resistance for Seed 1, while other seeds exhibit deviations that are not statistically significant. Variance components reveal considerable variability among subjects (variance = 80116.0).

TABLE S10
MIXED-EFFECTS MODEL RESULTS FOR RESISTANCE IN THE LAD ARTERY
(NUMBER OF OBSERVATIONS: 42).

Parameter	Estimate	95% CI	p-value
Seed 1	1589.0 (intercept)	1370.6, 1807.4	< 0.001
Seed 2	16.4	-69.9, 102.8	0.709
Seed 3	35.9	-50.4, 122.3	0.414
Seed 4	20.5	-65.8, 106.9	0.640
Seed 5	0.5	-85.8, 86.9	0.990
Seed 6	23.4	-62.9, 109.8	0.594

Table S11 presents the results of the mixed-effects model for blood flow resistance in the left circumflex (LCx) artery. The intercept signifies the baseline resistance for Seed 1, while the other seeds exhibit deviations that are not statistically significant. Variance components reveal considerable variability among subjects (variance = 90653.4).

TABLE S11
MIXED-EFFECTS MODEL RESULTS FOR RESISTANCE IN THE LCX ARTERY
(NUMBER OF OBSERVATIONS: 42).

Parameter	Estimate	95% CI	p-value
Seed 1	2240.8 (intercept)	2008.5, 2473.1	< 0.001
Seed 2	-15.232	-107.0, 76.5	0.745
Seed 3	-12.881	-104.6, 78.9	0.783
Seed 4	-4.537	-96.3, 87.2	0.923
Seed 5	2.258	-89.5, 94.1	0.962
Seed 6	-32.419	-124.2, 59.4	0.489

Table S12 presents the results of the mixed-effects model for blood flow resistance in the right coronary artery (RCA). The intercept denotes the baseline resistance for Seed 1, while the deviations observed in other seeds are statistically insignificant. Variance components reveal considerable variability among subjects (variance = 82431.3).

TABLE S12
MIXED-EFFECTS MODEL RESULTS FOR RESISTANCE IN THE RCA ARTERY
(NUMBER OF OBSERVATIONS: 42).

Parameter	Estimate	95% CI	p-value
Seed 1	1979.7 (intercept)	1758.2, 2201.2	< 0.001
Seed 2	6.147	-81.4, 93.8	0.891
Seed 3	21.433	-66.2, 109.1	0.632
Seed 4	55.316	-32.3,142.9	0.216
Seed 5	-5.511	-93.1, 82.1	0.902
Seed 6	15.301	-72.3, 102.9	0.732

Table S13 presents the results of the mixed-effect model analyzing Dynamic Time Warping (DTW) distances within the LAD artery. The model evaluates DTW distances across various seed combinations in this artery. The intercept (0.438, $p<0.001$) denotes the baseline DTW distance for Seed 1. The fixed effects associated with other seeds do not demonstrate significant deviations from this baseline, as none of their coefficients reach statistical significance ($p>0.05$). The variance attributed to the group level (0.042) reflects moderate variability among groups, whereas Seed 1 exhibits some variability (variance = 0.010), indicating potential differences in DTW distances across different seed combinations.

TABLE S13
MIXED-EFFECTS MODEL RESULTS FOR DTW DISTANCES IN THE LAD ARTERY
(NUMBER OF OBSERVATIONS: 105).

Parameter	Estimate	95% CI	p-value
Seed 1	0.438 (intercept)	0.026, 0.636	< 0.001
Distance: Seed 2 – Seed 1	0.018	-0.109, 0.145	0.777
Distance: Seed 3 – Seed 1	-0.026	-0.158, 0.106	0.697
Distance: Seed 4 – Seed 1	-0.046	-0.186, 0.095	0.524
Distance: Seed 5 – Seed 1	-0.093	-0.255, 0.070	0.262
Distance: Seed 6 – Seed 1	0.018	-0.109, 0.145	0.785

Table S14 presents the results of the mixed-effects model for DTW distances within the LCx artery. In this context, the intercept (0.399, p=0.005) denotes the baseline DTW distance for the reference seed (Seed 1). Although the coefficients associated with other seeds imply variations, none reach statistical significance (p>0.05). The group-level variance (0.082) suggests variability among subjects, with a notable variance in seed combinations (variance = 0.038). This model indicates marginally higher group variability relative to the LAD; however, the fixed effects do not attain significance.

TABLE S14
MIXED-EFFECTS MODEL RESULTS FOR DTW DISTANCES IN THE LCX ARTERY
(NUMBER OF OBSERVATIONS: 105).

Parameter	Estimate	95% CI	p-value
Seed 1	0.399 (intercept)	0.119, 0.680	0.005
Distance: Seed 2 – Seed 1	-0.096	-0.316, 0.124	0.392
Distance: Seed 3 – Seed 1	-0.090	-0.314, 0.134	0.430
Distance: Seed 4 – Seed 1	-0.141	-0.371, 0.090	0.231
Distance: Seed 5 – Seed 1	-0.189	-0.436, 0.058	0.133
Distance: Seed 6 – Seed 1	0.081	-0.057, 0.218	0.251

Table S15 presents the results of the mixed-effects model for DTW distances in the RCA artery. The intercept of the RCA artery model (0.449, p=0.001) signifies the baseline DTW distance for Seed 1. None of the fixed effects for other seeds attain statistical significance (p>0.05). Variance components indicate moderate variability among groups (variance = 0.076), with smaller contributions observed from seed combinations. The model implies that DTW distances across seeds in the RCA are relatively consistent.

TABLE S15
MIXED-EFFECTS MODEL RESULTS FOR DTW DISTANCES IN THE RCA ARTERY
(NUMBER OF OBSERVATIONS: 105).

Parameter	Estimate	95% CI	p-value
Seed 1	0.449 (intercept)	0.189, 0.709	0.001
Distance: Seed 2 – Seed 1	-0.077	-0.231, 0.077	0.325
Distance: Seed 3 – Seed 1	0.015	-0.144, 0.134	0.856
Distance: Seed 4 – Seed 1	-0.037	-0.204, 0.173	0.660
Distance: Seed 5 – Seed 1	-0.002	-0.190, 0.129	0.983
Distance: Seed 6 – Seed 1	-0.060	-0.225, 0.105	0.479

Tables S16 and **S17** illustrate the relationship between synthetic flow values (SBFP) across six seeds and the reference flow variable (fMBV flow rate), accounting for myocardial segment location (base, mid, and apex) and subject-specific variability. The intercept (swine: 0.347, p<0.001; volunteers: -0.065, p=0.048) indicates that, at baseline, synthetic flow slightly deviates from the reference flow, suggesting a minor systematic bias. The fMBV flow rate (swine: coefficient=0.926, volunteers: coefficient=0.978, p<0.001) demonstrates a nearly one-to-one relationship with synthetic SBFP, indicating strong alignment between synthetic and FE-MRI fMBV-derived flow rates. Significant effects were observed for myocardial segments in swine subjects; the basal (coefficient=0.433, p<0.001) and mid (coefficient=0.205, p<0.001) segments exhibited higher synthetic SBFP compared to the reference (apical) segment, suggesting flow variation across myocardial regions. A similar trend was observed in human volunteers. The fixed effects for the seeds were not statistically significant, with coefficients close to zero (e.g., volunteer Seed 2: 0.000, p=0.994), indicating minimal variation in synthetic flow across different seeds. Random effects indicate minor variability between subjects (volunteer subject variance = 0.001) and negligible variability attributed to the seeds, suggesting that synthetic flow remains consistent across subjects and seeds. Overall, the model affirms a strong correlation between synthetic SBFP and reference FE-MRI fMBV-derived flow rates, while also identifying myocardial segment-specific differences.

TABLE S16

MIXED-EFFECTS MODEL RESULTS FOR SBFP (NUMBER OF OBSERVATIONS: 960) AND ITS AGREEMENT WITH FE-MRI FMBV-DERIVED FLOW RATE PERCENTAGES FOR HUMAN VOLUNTEERS. FIXED EFFECTS CAPTURE THE DEVIATIONS FOR SEEDS (SEED 2–SEED 6).

Parameter	Estimate	95% CI	p-value
Seed 1	-0.065 (intercept)	-0.12, -0.001	0.048
Seed 2	~0	-0.033, 0.034	0.994
Seed 3	~0	-0.034, 0.034	0.993
Seed 4	~0	-0.034, 0.034	0.992
Seed 5	~0	-0.034, 0.034	0.992
Seed 6	~0	-0.034, 0.034	0.992
Basal segment	0.369	0.343, 0.395	< 0.001
Mid segment	0.254	0.228, 0.279	< 0.001
fMBV flow rate	0.978	0.973, 0.984	< 0.001

TABLE S17

MIXED-EFFECTS MODEL RESULTS FOR SBFP (NUMBER OF OBSERVATIONS: 684) AND ITS AGREEMENT WITH FE-MRI FMBV-DERIVED FLOW RATE PERCENTAGES FOR SWINE SUBJECTS. FIXED EFFECTS CAPTURE THE DEVIATIONS FOR SEEDS (SEED 2–SEED 6).

Parameter	Estimate	95% CI	p-value
Seed 1	0.347 (intercept)	0.200, 0.493	< 0.001
Seed 2	$<10^{-4}$	-0.077, 0.077	~1.0
Seed 3	$<10^{-4}$	-0.077, 0.077	~1.0
Seed 4	$<10^{-4}$	-0.077, 0.077	~1.0
Seed 5	$<10^{-4}$	-0.078, 0.078	~1.0
Seed 6	$<10^{-4}$	-0.078, 0.078	~1.0
Basal segment	0.433	0.371, 0.495	< 0.001
Mid segment	0.205	0.147, 0.262	< 0.001
fMBV flow rate	0.926	0.910, 0.943	< 0.001

S6. References

1. Huo, Y.; Kassab, G.S. Pulsatile Blood Flow in the Entire Coronary Arterial Tree: Theory and Experiment. *American Journal of Physiology-Heart and Circulatory Physiology* **2006**, *291*, H1074–H1087, doi:10.1152/ajpheart.00200.2006.
2. Kim, H.J.; Vignon-Clementel, I.E.; Coogan, J.S.; Figueroa, C.A.; Jansen, K.E.; Taylor, C.A. Patient-Specific Modeling of Blood Flow and Pressure in Human Coronary Arteries. *Ann Biomed Eng* **2010**, *38*, 3195–3209, doi:10.1007/s10439-010-0083-6.
3. Duanmu, Z.; Chen, W.; Gao, H.; Yang, X.; Luo, X.; Hill, N.A. A One-Dimensional Hemodynamic Model of the Coronary Arterial Tree. *Front. Physiol.* **2019**, *10*, 853, doi:10.3389/fphys.2019.00853.
4. Talou, G.D.M.; Safaei, S.; Hunter, P.J.; Blanco, P.J. Adaptive Constrained Constructive Optimisation for Complex Vascularisation Processes. *Sci Rep* **2021**, *11*, 6180, doi:10.1038/s41598-021-85434-9.
5. OLUFSEN, M.S.; HILL, N.A.; VAUGHAN, G.D.A.; SAINSBURY, C.; JOHNSON, M. Rarefaction and Blood Pressure in Systemic and Pulmonary Arteries. *J Fluid Mech* **2012**, *705*, 280–305, doi:10.1017/jfm.2012.220.
6. Pries, A.R.; Secomb, T.W.; Gessner, T.; Sperandio, M.B.; Gross, J.F.; Gaehtgens, P. Resistance to Blood Flow in Microvessels in Vivo. *Circulation Research* **1994**, doi:10.1161/01.RES.75.5.904.
7. Hutchins, G.M.; Miner, M.M.; Boitnott, J.K. Vessel Caliber and Branch-Angle of Human Coronary Artery Branch-Points. *Circ Res* **1976**, *38*, 572–576, doi:10.1161/01.res.38.6.572.
8. Messenger, J.C.; Chen, S.Y.; Carroll, J.D.; Burchenal, J.E.; Kioussopoulos, K.; Groves, B.M. 3D Coronary Reconstruction from Routine Single-Plane Coronary Angiograms: Clinical Validation and Quantitative Analysis of the Right Coronary Artery in 100 Patients. *Int J Card Imaging* **2000**, *16*, 413–427, doi:10.1023/a:1010643426720.
9. Brinkman, A.M.; Baker, P.B.; Newman, W.P.; Vigorito, R.; Friedman, M.H. Variability of Human Coronary Artery Geometry: An Angiographic Study of the Left Anterior Descending Arteries of 30 Autopsy Hearts. *Ann Biomed Eng* **1994**, *22*, 34–44, doi:10.1007/BF02368220.
10. Kassab, G.S.; Rider, C.A.; Tang, N.J.; Fung, Y.C. Morphometry of Pig Coronary Arterial Trees. *American Journal of Physiology-Heart and Circulatory Physiology* **1993**, *265*, H350–H365, doi:10.1152/ajpheart.1993.265.1.H350.
11. Kloska, M.; Grmanova, G.; Rozinajova, V. Expert Enhanced Dynamic Time Warping Based Anomaly Detection. *Expert Systems with Applications* **2023**, *225*, 120030, doi:10.1016/j.eswa.2023.120030.
12. Virtanen, P.; Gommers, R.; Oliphant, T.E.; Haberland, M.; Reddy, T.; Cournapeau, D.; Burovski, E.; Peterson, P.; Weckesser, W.; Bright, J.; et al. SciPy 1.0: Fundamental Algorithms for Scientific Computing in Python. *Nat Methods* **2020**, *17*, 261–272, doi:10.1038/s41592-019-0686-2.
13. Seabold, S.; Perktold, J. Statsmodels: Econometric and Statistical Modeling with Python.; Austin, Texas, 2010; pp. 92–96.

14. Giorgino, T. Computing and Visualizing Dynamic Time Warping Alignments in *R* : The **Dtw** Package. *J. Stat. Soft.* **2009**, *31*, doi:10.18637/jss.v031.i07.
